# Patterns of social inequality in arts and cultural participation: Findings from a nationally representative sample of adults living in the United Kingdom of Great Britain and Northern Ireland

**Published:** 2020-03-30

**Authors:** Hei Wan Mak, Rory Coulter, Daisy Fancourt

**Affiliations:** 1 Department of Behavioural Science and Health, University College London, England; 2 Department of Geography, University College London, London, England

**Keywords:** Arts And Cultural Participation, Latent Class Analysis, Social And Health Inequalities

## Abstract

**Context:**

A significant amount of literature indicates the health benefits of arts engagement. However, as this engagement is socially patterned, differential access to and participation in the arts may contribute to social and health inequalities.

**Objective:**

This study aimed to uncover the patterns of participation in arts activities and engagement with culture and heritage among adults in the United Kingdom of Great Britain and Northern Ireland, and to examine whether such patterns are associated with demographic and socioeconomic characteristics.

**Methodology:**

We applied latent class analysis to data on arts and cultural participation among 30 695 people in the Understanding Society study. Multinomial logistic regression was used to identify predictors for the patterns of activity engagement.

**Results:**

For arts participation, adults were clustered into “engaged omnivores,” “visual and literary arts,” “performing arts” and “disengaged.” For cultural engagement, adults were clustered into “frequently engaged,” “infrequently engaged” and “rarely engaged.” Regression analysis showed that the patterns of arts activity were structured by demographic and socioeconomic factors.

**Conclusion:**

This study reveals a social gradient in arts and cultural engagement. Given the health benefits of arts engagement, this suggests the importance of promoting equal access to arts and cultural programmes, to ensure that unequal engagement does not exacerbate health inequalities.

## Background

The 2019 WHO Health Evidence Synthesis Report on arts and health identified over 3000 studies on the mental and physical health impacts of engaging with the arts (1). However, arts and cultural engagement is unevenly patterned. This was notably described by Bourdieu who theorised that monetary resources and cultural exclusion enable and constrain participation in arts and cultural activities, and thus (re)produce social inequality (2). Indeed, over the past few years, several reports from arts and governmental organizations have shown how patterns of arts engagement differ based on socioeconomic factors such as socioeconomic status (SES) and parental education and income (3–5). This social patterning is important, as the same patterning underlies inequalities in health (6). Consequently, it has been proposed that differential access to and participation in the arts may contribute to social and health inequalities (7).

However, there are limitations to the existing evidence on inequalities in arts engagement. Existing studies have not always used representative samples, have generally focused on individual socioeconomic factors rather than simultaneously considering multiple factors that may be related to one another, and have focused on indices of overall engagement or on a priori selected categories of engagement. These a priori categories may conflate diverse activities with different types of social patterning. Understanding patterns of arts engagement is therefore important, both to get a better sense of how people engage in different types of activities, and to develop a more nuanced understanding of how inequalities might both contribute to and result from engagement with the arts.

Therefore, in this paper, we used a large nationally representative sample of adults in the United Kingdom to (i) identify latent categories of participation in arts activities (e.g. drawing, singing, playing a musical instrument) and engagement with culture and heritage (e.g. visiting museums or exhibitions, attending concerts, visiting historical sites), and (ii) identify how patterns of engagement vary depending on a rich panel of demographic and socioeconomic factors.

## Data And Methods

Data were drawn from Understanding Society, a nationally representative longitudinal study, which is a continuation of the British Household Panel Survey (BHPS) started in 1991 (8). Understanding Society annually interviews approximately 50 000 individuals from 40 000 households in the United Kingdom. The survey covers a wide range of topics including education, employment, social engagement and health. The Wave 2 (2010–2012) and Wave 5 (2013–2015) interviews captured participation in arts and cultural activities. To ensure a large sample size, this study used the Wave 2 data that contain 38 069 participants. In our analysis, we included participants who provided full data across all measures (*n* = 30 695).

### Measurements

Understanding Society contains a particularly rich set of variables on arts participation and cultural engagement. In our analyses, we focused on non-digital and non-religious-specific arts and cultural activities. For arts participation, we considered 9 variables: dancing; singing to an audience or rehearsing for a performance (not karaoke); playing a musical instrument; rehearsing/performing in a play/drama, opera/operetta or musical theatre; taking part in a carnival/street arts event (e.g. as a musician, dancer or costume maker); painting, drawing, printmaking or sculpture; taking part in textile crafts, wood crafts or any other crafts, such as embroidery and knitting; reading for pleasure (not newspapers, magazines or comics); and writing any stories, plays or poetry. Respondents were asked whether they had engaged in each of these activities in the past 12 months.

For cultural engagement, we considered a set of 14 variables: being a member of a book club or attending an event connected with books or writing; visiting an exhibition or collection of art, photography, sculpture or a craft exhibition (not a craft market); attending a play/drama, pantomime or musical; going to an opera/operetta or a classical music performance; going to a rock, pop or jazz performance; going to a ballet or contemporary dance performance; visiting a museum; visiting a city or town with historic character; visiting a historic building (non-religious) open to the public; visiting a place connected with industrial history (e.g. an old factory, dockyard or mine) or historic transport system (e.g. an old ship or railway); visiting a historic place of worship as a visitor (not as a worshiper); visiting a monument (e.g. a castle, fort or ruin); visiting a site of archaeological interest (e.g. a Roman villa or ancient burial site); and visiting a site connected with sports heritage (e.g. Wimbledon), but not for the purpose of watching a sport. Again, respondents were asked whether they had been to each of the events/heritage locations in the past 12 months.

To understand social patterns of arts participation, we considered a set of demographic and socioeconomic predictors. Demographic factors included respondents’ age, gender and ethnicity (White vs Asian/Asian British vs Black/Black British vs Mixed or Others), whether respondents were living alone, partnership status (single and never married vs married or in cohabitation vs separated or divorced or widowed), and whether respondents were responsible for children under the age of 16.

Socioeconomic factors included respondents’ educational levels (university degree vs advanced (higher degree/A-level) vs General Certificate of Secondary Education (GCSE) or equivalent vs other/no qualification), current occupational socioeconomic status (SES) (managerial/professional vs intermediate/small employment or own account vs lower supervisory/lower technical/semi-routine or routine vs unemployed (defined as not working in the past week, looking for jobs in the past month and able to start work in 2 weeks)/retired/full time student/others (e.g. looking after family)), the SES of the respondents’ parents when the respondent was 14 years old (managerial/professional vs intermediate/small employers/own account vs lower supervisory/lower technical/semi-routine or routine vs none of the parents work), logged monthly household income, and housing tenure (house owner vs social rent vs private rent).

### Statistics

To understand patterns of arts participation and cultural engagement, latent class analysis (LCA) was applied to estimate the probabilities of respondents belonging to a given class and to assign respondents to different arts participation and cultural engagement groups. LCA is a technique that observes the relationship between a set of unobserved variables and identifies a set of mutually exclusive latent classes within a population (9). One advantage of LCA is that it reduces the bias caused by traditional arbitrary cutting point methods when categorising participants into different memberships. Instead, categories are derived inductively, with the most effective and parsimonious model solution (defined using the goodness of fit statistical test, *P*-value of the likelihood-ratio (G^2^) statistic that is larger than 0.05, and the smaller values of Akaike information criterion (AIC) and Bayesian information criterion (BIC)) being selected.

Arts participation and cultural engagement variables were inputted to the LCA with all indicators coded as binary variables. As data sparseness can create empty cells and thus unreliable analysis (10), we dropped the uncommon category of taking part in a carnival or street arts event (in which only 2.66% of the sample reported having taken part).

We compared the results for 3-, 4- and 5-class models. For each class model, we omitted one activity to test whether the model improved based on the goodness of fit test. We repeated this procedure until the model would no longer converge. We then identified the best fitting latent class model for the data that satisfied the goodness of fit test requirement and for which classes and class probabilities were interpretable (11).

For arts participation, the model fit indexes suggest that a 4-class LCA model with a set of five indicators had the best result (G^2^ = 15.343, residual degree of freedom = 8, *P*-value = 0.053, AIC = 93441.833, BIC = 93638.418). The five indicators included singing to an audience or rehearsing for a performance (not karaoke); playing a musical instrument; rehearsing or performing in a play/drama, opera/operetta or musical theatre; painting, drawing, printmaking or sculpture; and writing stories, plays or poetry. The latent classes represent 1.18%, 4.54%, 4.63% and 89.7%, respectively, of the sample. According to the conditional probability of the arts activities, class 1 participants had the highest levels of all arts activities engagement and were therefore labelled as “engaged omnivores.” Class 2 participants had lower levels of arts engagement and tended to engage more in creative arts activities such as painting, drawing, and writing stories than performing arts activities; they were therefore labelled as the “visual and literary arts” class. Conversely, class 3 participants had higher levels of engaging in performing or musical activities, such as singing to an audience, playing a musical instrument and performing in a play/drama, than creative arts activities. They were therefore referred to as the “performing arts” class. Class 4 participants had very low levels of engagement in any activities and were labelled as “disengaged” (see Table 1).

For cultural engagement, a different pattern of this type of arts participation was found. The model fit statistics suggest that a 3-class LCA model with the set of 14 indicators had the best result (G^2^ = 16244.232, residual degree of freedom = 16339, *P*-value = 0.699, AIC = 421400.397, BIC = 421776.472). According to the conditional probability of cultural engagement, there was no difference in patterns of engagement in some activities, but the three classes did represent different frequencies of participation: “frequently engaged” (18.4%), “infrequently engaged (33.9%) and “rarely engaged” (47.7%) (Table 2).

To understand the associations between arts participation and cultural engagement and demographic backgrounds and socioeconomic characteristics, we used multinomial logistic regression. All analyses were weighted using the weights supplied by the survey administrators to account for nonresponse and to ensure our estimates were representative. Our models produced the relative risk ratio (RRR), showing the percentage “relative risk” that an individual would be categorised as a particular type of engager (e.g. engaged omnivore) for each demographic and socioeconomic factor, while adjusting for all other demographic and socioeconomic factors in the model. An RRR of higher than 1 implies a higher likelihood of being a particular type of engager relative to the omitted baseline group, while an RRR of lower than 1 implies a lower likelihood. Given that arts participation and cultural engagement patterns are likely to be related at the household level, the 95% confidence interval in regression models is calculated by clustering standard errors within households. All statistical models met model assumptions with analyses carried out in Stata v15 and R Studio.

## Results

In our sample, the average age was 48 years (SD = 18.4). Fifty-five % were female and 91% were white. On average, 65% of the sample were married, 35% had a university degree and 26% were in managerial/professional occupations (Table 3). The distribution of arts participation and cultural engagement groups by demographic backgrounds and socioeconomic characteristics is shown in Table 4 and Table 5. The cross-tabulation between patterns of arts participation and cultural engagement is shown in Table 6.

### Arts Participation

#### Demographics

Using the “disengaged” as the reference group, respondents who were older had a lower RRR of being an “engaged omnivore” or engaging in visual and literary arts, but age was not related to an RRR of engaging in performing arts (Table 7). Women had a 32% higher RRR of engaging in performing arts. Compared to people of white ethnicity, people who were Asian/Asian British had a lower RRR of engaging in any arts activities, while people who were Black/Black British had a 62% higher RRR of engaging in performing arts. Living alone was not related to arts engagement, but people who were not married had a higher RRR of engaging in all arts activities. Being responsible for children under the age of 16 was associated with a 48% lower RRR of being an engaged omnivore and a 21% and 41% lower RRR of engaging in visual and literary or performing arts, respectively.

#### Socioeconomic characteristics

Compared to individuals with a university degree, those with fewer educational qualifications had a lower RRR of engaging in any arts activities (Table 7). Those with no qualifications had an 80% lower RRR of being engaged omnivores, an 84% lower RRR of engaging in visual and literary arts, and a 72% lower RRR of engaging in performing arts. Individuals in lower supervisory or lower technical jobs had a 52% lower RRR of being an engaged omnivore compared to those in managerial/professional roles, while individuals who were unemployed had a 50% higher RRR of engaging in visual and literary arts activities. But otherwise, socioeconomic status was not related to arts engagement.

However, parents’ SES at age 14 was more clearly related, with individuals whose parents worked in intermediate or lower supervisory jobs having a lower RRR of most arts engagement. Household income only showed a modest relationship to arts participation (14% lower RRR of being an engaged omnivore for each descending income log). Compared to those who owned their own homes, those in social housing had a 41% lower RRR of engaging in performing arts, while those renting privately had a 45% higher RRR of being arts omnivores and a 46% higher RRR of engaging in visual and literary arts.

### Cultural Engagement

#### Demographics

Using the “rarely engaged” group as the reference group, respondents who were older had a higher RRR of engaging frequently in cultural activities, with women having a 9% higher RRR of being infrequently engaged and frequently engaged (Table 8). People who were non-white had a lower RRR of being engaged in cultural activities with any level of frequency, with the RRR being most pronounced for those who were Asian or Black (both 86% lower RRR) rather than of mixed ethnicity (28% lower RRR). Living alone was related to a 15% and 21% higher RRR of infrequent or frequent engagement, respectively. But being unmarried (either through being single or divorced/widowed) was associated with a lower RRR of engagement. People with children under the age of 16 had a 12% lower RRR of engaging infrequently and a 35% lower RRR of engaging frequently.

#### Socioeconomic characteristics

Compared to individuals with a university degree, those with fewer educational qualifications had a lower RRR of being culturally engaged, both infrequently and frequently (Table 8). Compared with individuals in managerial or professional roles, those of intermediate, lower supervisory roles or those who were unemployed had a lower RRR of being cultural engaged, both infrequently and frequently. The same pattern was observed for parental SES, but the RRR were less marked. Household income was related to greater engagement, with a 21% higher RRR of being infrequently engaged for each income log, and a 73% higher RRR of being frequently engaged. Finally, compared to individuals who owned their own homes, those in social housing had a 49% lower RRR of being infrequently engaged and a 67% lower RRR of being frequently engaged, while those renting privately had a 20% lower RRR of being infrequently engaged.

## Discussion

This study is one of the first to explore the patterns of arts participation and cultural engagement across the United Kingdom and to examine how the patterns are associated with individuals’ demographic and socioeconomic characteristics. Our results demonstrate that, in addition to there being demographic factors that predict engagement both in participatory arts activities and with culture and heritage, there is a clear social gradient across participation. Although there are few existing empirical studies on the determinants of arts and cultural activity participation, our findings echo previous research and reports (3–5) on social patterns of participation in arts and cultural engagement.

Many of the demographic predictors of participation were the same for both arts and cultural activities. For example, there was higher engagement in both types of activities among women than men, which has been discussed extensively in sociological literature (12, 13). There was also lower engagement among those who were responsible for children under the age of 16, most likely due to time constraints. However, there were also some notable differences between predictors of engagements in arts and culture. For example, while being married was associated with lower arts participation, it was also associated with higher cultural engagement. This finding suggests that arts and cultural engagement present different types of exposure and that different interpersonal factors may influence individuals’ motivation to engage in participatory arts activities and with culture and heritage. Another difference in predictors between arts and cultural engagement was for ethnicity. Although there was some evidence of lower engagement in arts by ethnic minorities, this was limited to individuals of Asian/Asian British ethnicity (in line with a recent report from the United Kingdom Department for Digital, Culture, Media and Sport (14)), while Black/British respondents reported higher engagements in performing arts. However, for cultural engagement, there was lower engagement across all non-white ethnic groups. It is possible that non-white ethnic minorities are overrepresented in lower SES groups (15), which in turn may explain their lower cultural engagements, but notably, these findings were independent of socioeconomic factors, so this cannot be the sole explanatory factor. Research has suggested the lower engagement in arts among Asian groups may be due both to the cost of participating or attending but also due to concerns about feeling uncomfortable (16), suggesting that a perceived lack of individual cultural resonance or perceived stigma may also play a role in the differences found here.

For socioeconomic predictors, there was a combination of common patterns and divergences between arts and cultural engagement. In terms of commonality, education was associated with higher engagement in both activities. Education could increase cognitive capacity to engage, increase awareness of activities, provide cultural and historical references that support enjoyment from engaging (5), and help to cultivate cultural tastes and preferences (17). SES and parental SES were also predictors of both arts and cultural engagement, but SES was a much stronger predictor of cultural engagement while parental SES was a stronger predictor of arts participation. It has been suggested that class of origin continues to matter throughout the lifespan since individuals from higher SES family backgrounds can benefit from their parents’ resources (e.g. cultural capital) (2). In relation to why we found this to be stronger than individual SES for arts, it is also possible that childhood exposure plays an important role. Many participatory arts activities require training or experience to fully benefit from participation (e.g. learning to play an instrument or cultivating a skill in drawing or needlework). Such activities are typically encouraged in childhood among those whose parents are of higher SES more than those whose parents are of lower SES (3). Therefore when children grow up, although their own SES may affect whether they learn a new participatory skill, their parents’ SES will affect whether they already possess skills and training in participatory arts activities.

With regards to economic factors, there was little evidence of a relationship between household income and arts participation, apart from a marginally lower engagement as an arts omnivore among those with higher income, while for cultural engagement, monthly income was a clear predictor of engagement. Given that most cultural engagement involves non-free activities (such as tickets for a ballet performance), monetary resources may be required to support these activities. Indeed, the significantly lower cultural engagement among individuals in social housing compared to those who owned properties suggests that financial factors play a key role in cultural engagement.

The findings that there are demographic and socioeconomic differences in arts and cultural engagement are important when considering that many of the predictors identified are also predictors of poor health outcomes. For example, men have a shorter life expectancy than women (18), while individuals of minority ethnic backgrounds (19), lower educational attainment (20), lower socioeconomic status (21), lower household income (21), and those living in social housing (22) are all associated with an increased risk of poor health outcomes. The similarities in the predictors of arts engagement and poor health could merely be because arts engagement presents a form of capital, similar to health, that can be attained by those with more material resources (e.g. money and employment) and non-material resources (e.g. a high sense of personal control, social support) (23). However, the other interpretation is to see arts and cultural engagement as modifiable risk factors, similar to other health behaviours such as exercise, smoking, alcohol consumption and substance use; factors that are not merely correlates of health but predictors of it. In this light, the differential participation in arts and culture by both demographic and socioeconomic factors is a cause for concern, as it could be exacerbating existing health inequalities (7). In support of this theory is the large and growing body of evidence regarding the health benefits of arts participation, both among the population as a whole but also specifically among minority groups, individuals of lower SES and individuals with poor health (24–26). Further, arts participation has been shown to affect health independent of demographic and socioeconomic factors (27), suggesting that arts participation is not simply a proxy for these factors.

Given the well evidenced health benefits of engaging with the arts, it is possible that encouraging arts and cultural engagement could help to improve health among disadvantaged populations and reduce health inequalities (28). With this in mind, future research needs to focus on understanding why engagement is lower among different demographic and socioeconomic groups, such as families, individuals from minority backgrounds, individuals of lower educational attainment and individuals of lower socioeconomic status. In particular, such research needs to identify what the barriers and enablers of participation are, and to identify and test interventions to promote engagement, as well as measure whether increased engagement is associated with a lower risk of adverse health outcomes.

## Conclusions

Overall, this study identified different patterns for arts participation and cultural engagement across society in the United Kingdom and found that these patterns are closely associated with demographic and socioeconomic characteristics. While this study focused specifically on the United Kingdom, international studies have shown similar patterns across other high-income countries (29). This suggests that there is a social gradient in arts and cultural engagement that appears to be in parallel to the gradient in health, with the most privileged individuals enjoying more opportunities to engage in the arts, which in turn can further enhance health. Research suggests that arts and cultural participation are not merely correlates of health but may be modifiable health behaviours that could help to reduce inequalities in health. As a result, future studies are encouraged to promote and encourage arts and cultural engagement on a larger scale, particularly among those from minority and lower socioeconomic backgrounds. Equal access to arts and cultural programmes has, in theory, the potential to help reduce health inequalities through narrowing the gap of social and cultural capital between the advantaged and disadvantaged populations. Future studies, including qualitative and mixed methods analyses of participation in the arts across population subgroups, are strongly encouraged to test whether this is indeed the case.

## Figures and Tables

**Table 1:** Estimated size of the latent classes and probability of arts participation in % (*N* = 38 069)

	Engaged omnivore	Visual and literary arts	Performing arts	Disengaged	Overall
**Estimated size**	**1.18**	**4.54**	**4.63**	**89.7**	**100**
Sang to an audience or rehearsed for a performance (not karaoke)	73.9	0.00	63.0	0.72	5.25
Played a musical instrument	58.9	25.1	46.3	3.87	9.60
Rehearsed or performed in a play/ drama, opera/operetta or musical theatre	56.3	3.27	22.7	0.35	2.51
Painting, drawing, printmaking or sculpture	59.6	54.9	25.9	7.10	15.6
Written any stories, plays or poetry	74.0	23.0	5.36	1.49	5.50

Note: Goodness of fit statistics of the 4-class model: likelihood-ratio (G^2^) test = 15.343; residual degree of freedom = 8; p-value = 0.053; AIC = 93441.833; BIC = 93638.418. Goodness of fit statistics of the 3-class model: likelihood-ratio (G^2^) test = 153.072; residual degree of freedom = 14; p-value = 0.000; AIC = 93567.562; BIC = 93712.863. Goodness of fit statistics of the 5-class model: likelihood-ratio (G^2^) test = 40.875; residual degree of freedom = 5; p-value = 0.000; AIC = 93473.365; BIC = 93695.591.

**Table 2:** Estimated size of the latent classes and probability of cultural engagement in % (*N* = 38 069)

	Frequently engaged	Infrequently engaged	Rarely engaged	Overall
**Estimated size**	**18.4**	**33.9**	**47.7**	**100**
Been a member of a book club, where people meet up to discuss and share books/been to an event connected with books or writing	25.4	6.81	1.39	7.68
Been to an exhibition or collection of art, photography or sculpture or a craft exhibition (not craft market)	78.4	31.4	2.79	26.6
Been to a play/drama, pantomime or a musical	74.3	43.7	12.6	34.7
Been to an opera/operetta or a classical music performance	35.2	9.38	1.51	10.4
Been to a rock, pop or jazz performance	48.8	30.2	8.76	23.5
Been to ballet or contemporary dance	20.2	6.37	1.21	6.50
Visited museums	90.0	52.1	8.22	44.9
Visited a city or town with historic character	96.9	64.8	9.89	33.1
Visited a historic building open to the public (non-religious)	93.4	43.2	2.08	17.4
Visited a place connected with industrial history (e.g. an old factory, dockyard or mine) or historic transport system (e.g. an old ship or railway)	52.6	20.4	1.40	23.8
Visited a historic place of worship attended as a visitor (not to worship)	75.2	25.8	2.26	32.8
Visited a monument such as a castle, fort or ruin	85.1	44.4	3.91	13.1
Visited a site of archaeological interest (e.g. Roman villa, ancient burial site)	49.9	10.8	0.39	4.98
Visited a site connected with sports heritage (e.g. Wimbledon) (not visited for the purposes of watching sport)	13.9	5.98	0.75	38.4

Note: Goodness of fit statistics of the 3-class model: likelihood-ratio (G^2^) test = 16244.232; residual degrees of freedom = 16339; p-value = 0.699; AIC = 421400.397; BIC = 421776.472. Goodness of fit statistics of the 4-class model: likelihood-ratio (G^2^) test = 12855.579; residual degree of freedom = 16324; p-value = 1.000; AIC = 418041.744; BIC = 418546.026. Goodness of fit statistics of the 5-class model: likelihood-ratio (G^2^) test = 11062.020; residual degree of freedom = 16309; p-value = 1.000; AIC = 416278.185; BIC = 416910.675

**Table 3:** Descriptive Statistics (*N* = 30 695)

Demographic backgrounds	%
Age (mean)	47.8
**Gender**	
Female	54.7
Male	45.3
**Ethnicity**	
White	91.4
Asian/Asian British	4.95
Black/Black British	2.09
Mixed/other	1.53
**Living alone**	
Yes	14.7
No	85.3
**Partnership status**	
Single and never married	21.0
Married or in cohabitation	64.9
Separated or divorced or widowed	14.1
**Responsible for child(ren) under age 16**	
Yes	16.5
No	83.5
**Socioeconomic characteristics**	**%**
**Educational levels**	
University degree	34.6
Advanced (higher degree/A-level)	19.6
GCSE or equivalent	20.7
Other/no qualification	25.1
**Socioeconomic status (SES)**	
Managerial/professional	26.1
Intermediate/small employment/own account	15.1
Lower supervision or lower technical/semi-routine or routine	22.4
Unemployed (incl. retired, full-time student)	36.3
**Parents’ SES at aged 14**	
Managerial/professional	26.2
Intermediate/small employers or own account	22.4
Lower supervisory or technical/semi-routine or routine	44.6
None of the parents work	6.82
**Household income monthly (quartile)**	
£0–£1 017	22.6
£1 018–£1 547	24.6
£1 548–£2 363	25.8
£2 364–£35 786	27.0
**Housing tenure**	
House owner	70.0
Social rent	17.1
Private rent	12.9

**Table 4:** Distribution of arts participation groups by demographic backgrounds and socioeconomic characteristics in % (*N* = 30 695)

	Engaged omnivore	Visual and literary arts	Performing arts	Disengaged	Total
*Demographic backgrounds*					
Age (mean)	37.1	38.7	45.4	48.5	47.8
Gender					
Female	46.6	53.7	57.3	54.7	54.7
Male	53.4	46.3	42.7	45.3	45.3
Ethnicity					
White	90.6	90.5	91.7	91.5	91.4
Asian/Asian British	3.40	4.08	3.42	5.10	4.95
Black/Black British	2.78	2.43	3.21	2.00	2.09
Mixed/other	3.18	2.99	1.66	1.43	1.53
Living alone					
Yes	14.2	14.2	15.0	14.7	14.7
No	85.8	85.8	85.0	85.3	85.3
Partnership status					
Single and never married	45.3	37.5	27.2	19.6	21.0
Married or in cohabitation	46.1	52.8	60.4	66.0	64.9
Separated or divorced or widowed	8.57	9.64	12.5	14.4	14.1
Responsible for child(ren) under 16					
Yes	10.6	16.7	12.7	16.8	16.5
No	89.5	83.3	87.3	83.2	83.5
*Socioeconomic characteristics*					
Educational levels					
University degree	57.7	53.7	50.6	32.5	34.6
Advanced (higher degree/A-level)	24.9	26.1	22.4	19.1	19.6
GCSE or equivalent	12.0	14.8	17.0	21.3	20.7
Other/no qualification	5.42	5.41	9.99	27.1	25.1
Socioeconomic status (SES)					
Managerial/professional	41.5	32.9	34.3	25.2	26.1
Intermediate/small employment/own account	13.6	15.3	16.7	15.1	15.1
Lower supervision or lower technical/semi-routine or routine	14.4	20.3	18.8	22.8	22.4
Unemployed (incl. retired, full-time student)	30.6	31.6	30.3	37.0	36.3
Parents’ SES at aged 14					
Managerial/professional	46.6	40.2	36.7	24.7	26.2
Intermediate/small employers or own account	21.0	21.5	26.0	22.3	22.4
Lower supervisory or technical/semi-routine or routine	26.0	31.9	32.2	46.1	44.6
None of the parents work	6.46	6.41	5.12	6.93	6.82
Household income monthly (quartile)					
£0–£1 017	22.3	21.4	16.2	23.0	22.6
£1 018–£1 547	18.7	22.2	20.4	25.0	24.6
£1 548–£2 363	24.9	24.2	26.4	25.9	25.8
£2 364–£35 786	34.1	32.2	37.0	26.1	27.0
Housing tenure					
House owner	63.9	63.1	76.2	70.1	70.0
Social rent	11.5	14.2	8.99	17.8	17.1
Private rent	24.6	22.8	14.8	12.1	12.9

**Table 5:** Distribution of cultural engagement groups by demographic backgrounds and socioeconomic characteristics in % (*N* = 30 695)

	Frequently engaged	Infrequently engaged	Rarely engaged	Total
*Demographic backgrounds*				
Age (mean)	48.5	46.3	48.7	47.8
Gender				
Female	51.4	55.0	56.0	54.7
Male	48.6	45.0	44.0	45.3
Ethnicity				
White	96.3	93.3	87.6	91.4
Asian/Asian British	1.68	3.86	7.38	4.95
Black/Black British	0.54	1.42	3.36	2.09
Mixed/other	1.48	1.40	1.66	1.53
Living alone				
Yes	13.6	12.9	16.8	14.7
No	86.4	87.1	83.2	85.3
Partnership status				
Single and never married	16.9	20.5	23.4	21.0
Married or in cohabitation	72.9	67.8	58.8	64.9
Separated or divorced or widowed	10.2	11.7	17.8	14.1
Responsible for child(ren) under 16				
Yes	12.6	17.6	17.5	16.5
No	87.4	82.4	82.5	83.5
*Socioeconomic characteristics*				
Educational levels				
University degree	61.5	39.1	18.3	34.6
Advanced (higher degree/A-level)	17.9	21.8	18.8	19.6
GCSE or equivalent	12.5	20.8	24.4	20.7
Other/no qualification	8.11	18.4	38.6	25.1
Socioeconomic status (SES)				
Managerial/professional	44.8	31.3	13.2	26.1
Intermediate/small employment/own account	15.7	17.2	13.2	15.1
Lower supervision or lower technical/semi-routine or routine	12.3	21.3	28.1	22.4
Unemployed (incl. retired, full-time student)	27.3	30.3	45.6	36.3
Parents’ SES at aged 14				
Managerial/professional	40.4	29.4	16.9	26.2
Intermediate/Small employers or own account	22.9	22.3	22.2	22.4
Lower supervisory or technical/semi-routine or routine	33.1	42.8	51.5	44.6
None of the parents work	3.61	5.51	9.39	6.82
Household income monthly (quartile)				
£0–£1 017	10.4	17.5	32.5	22.6
£1 018–£1 547	15.8	23.1	29.9	24.6
£1 548–£2 363	26.1	28.3	23.6	25.8
£2 364–£35 786	47.6	31.0	13.9	27.0
Housing tenure				
House owner	82.3	75.7	59.6	70.0
Social rent	4.65	11.6	27.5	17.1
Private rent	13.1	12.7	12.9	12.9

**Table 6:** Cross-Tabulation of arts participation against cultural engagement in % (*N* = 30 695)

		**Cultural engagement**			**Total**
		**Frequently engaged**	**Infrequently engaged**	**Rarely engaged**	
**Arts participation**	Engaged omnivore	3.48	1.01	0.16	1.14
	Visual and literary arts	9.19	4.78	1.97	4.45
	Performing arts	9.67	5.01	2.12	4.70
	Disengaged	77.7	89.2	95.8	89.7

Note: The ‘Total’ column shows the percentage of arts participation.

**Table 7:** Multinomial Logistic Regression Predicting Latent Class Membership For Arts Participation (*N* = 26 215)

	Engaged omnivore vs disengaged			Visual and literary arts vs disengaged			Performing arts vs disengaged		
	RRR	95%CI	*P*-value	RRR	95%CI	*P*-value	RRR	95%CI	*P*-value
*Demographic backgrounds*									
Age	0.98	0.97–0.99	0.000	0.98	0.97–0.98	0.000	1.00	0.99–1.01	0.960
Female^1^	0.86	0.65–1.13	0.269	1.04	0.89–1.21	0.664	1.32	1.15–1.50	0.000
Ethnicity^2^									
Asian/Asian British	0.35	0.19–0.66	0.001	0.51	0.39–0.66	0.000	0.56	0.42–0.75	0.000
Black/Black British	0.95	0.55–1.67	0.868	0.82	0.60–1.12	0.216	1.62	1.22–2.14	0.001
Mixed/other	1.21	0.65–2.26	0.547	1.22	0.86–1.74	0.271	0.96	0.61–1.51	0.847
Living alone	0.66	0.42–1.03	0.065	0.96	0.75–1.23	0.752	0.86	0.69–1.08	0.204
Partnership status^3^									
Single and never married	2.35	1.63–3.40	0.000	1.50	1.23–1.84	0.000	1.52	1.25–1.84	0.000
Separated or divorced or widowed	1.88	1.14–3.08	0.013	1.37	1.07–1.75	0.013	1.27	1.00–1.60	0.050
Responsible for child(ren) under age 16	0.52	0.34–0.81	0.003	0.79	0.65–0.96	0.016	0.59	0.48–0.72	0.000
*Socioeconomic characteristics*									
Educational levels^4^									
Advanced (higher degree/A–level)	0.57	0.40–0.81	0.001	0.66	0.55–0.79	0.000	0.77	0.65–0.91	0.003
GCSE or equivalent	0.33	0.21–0.50	0.000	0.38	0.31–0.46	0.000	0.58	0.48–0.70	0.000
Other/no qualification	0.20	0.11–0.37	0.000	0.16	0.12–0.21	0.000	0.28	0.22–0.36	0.000
Socioeconomic status (SES)^5^									
Intermediate/small employment or own account	0.71	0.46–1.10	0.128	1.02	0.82–1.28	0.855	1.02	0.83–1.26	0.828
lower supervision or lower technical/semi–routine or routine	0.48	0.32–0.74	0.001	0.95	0.76–1.19	0.640	0.88	0.72–1.08	0.222
Unemployed (incl. retired, fulltime student)	1.06	0.72–1.56	0.787	1.50	1.22–1.83	0.000	1.01	0.82–1.24	0.923
Parents’ SES at aged 14^5^									
Intermediate/small employers or own account	0.68	0.49–0.96	0.028	0.76	0.63–0.92	0.006	0.95	0.80–1.11	0.505
Lower supervisory or technical/semi–routine or routine	0.52	0.38–0.71	0.000	0.67	0.56–0.79	0.000	0.65	0.55–0.76	0.000
None of the parents work	0.74	0.39–1.41	0.361	0.79	0.59–1.05	0.102	0.76	0.54–1.07	0.114
Household income monthly (log)	0.86	0.75–1.00	0.046	0.98	0.90–1.06	0.569	1.00	0.89–1.13	0.944
Housing tenure^6^									
Social rent	0.83	0.49–1.40	0.476	1.02	0.82–1.28	0.847	0.59	0.47–0.76	0.000
Private rent	1.45	1.01–2.08	0.046	1.46	1.19–1.79	0.000	1.04	0.85–1.29	0.693
Constant	0.28	0.08–1.00	0.049	0.25	0.12–0.53	0.000	0.08	0.03–0.24	0.000
Pseudo R2	0.07								

Note: Reference groups are

1 Male

2 White

3 Married or in cohabitation

4 University degree

5 Managerial or professional

6 House owner.

**Table 8:** Multinomial Logistic Regression Predicting Latent Class Membership For Cultural Engagement (*N* = 26 215)

	Frequently vs rarely engaged			Infrequently vs rarely engaged		
	RRR	95%CI	*P*-value	RRR	95%CI	*P*-value
*Demographic backgrounds*						
Age	1.02	1.01–1.02	0.000	1.00	1.00–1.00	0.949
Female^1^	1.09	1.00–1.18	0.042	1.09	1.03–1.17	0.007
Ethnicity^2^						
Asian/Asian British	0.14	0.12–0.18	0.000	0.37	0.32–0.42	0.000
Black/Black British	0.14	0.10–0.20	0.000	0.39	0.32–0.46	0.000
Mixed/other	0.72	0.52–1.00	0.052	0.71	0.56–0.90	0.005
Living alone	1.21	1.02–1.44	0.028	1.15	1.01–1.31	0.038
Partnership status^3^						
Single and never married	0.85	0.73–1.00	0.045	0.83	0.74–0.94	0.002
Separated or divorced or widowed	0.64	0.54–0.76	0.000	0.75	0.66–0.85	0.000
Responsible for child(ren) under 16	0.65	0.57–0.74	0.000	0.88	0.80–0.97	0.008
*Socioeconomic characteristics*						
Educational levels^4^						
Advanced (higher degree/A–level)	0.44	0.39–0.50	0.000	0.68	0.61–0.75	0.000
GCSE or equivalent	0.25	0.22–0.29	0.000	0.53	0.48–0.58	0.000
Other/no qualification	0.10	0.09–0.11	0.000	0.33	0.30–0.37	0.000
Socioeconomic status (SES)^5^						
Intermediate/small employment or own account	0.68	0.59–0.77	0.000	0.76	0.68–0.85	0.000
Lower supervision or lower technical/semi-routine or routine	0.40	0.35–0.46	0.000	0.55	0.49–0.61	0.000
Unemployed (incl. retired, full–time student)	0.57	0.50–0.66	0.000	0.59	0.53–0.66	0.000
Parents’ SES at aged 14^5^						
Intermediate/small employers or own account	0.63	0.56–0.71	0.000	0.73	0.67–0.81	0.000
Lower supervisory or technical/semi–routine or routine	0.50	0.45–0.56	0.000	0.70	0.64–0.76	0.000
None of the parents work	0.45	0.37–0.55	0.000	0.63	0.55–0.73	0.000
Household income monthly (log)	1.73	1.57–1.91	0.000	1.21	1.14–1.29	0.000
Housing tenure^6^						
Social rent	0.33	0.27–0.39	0.000	0.51	0.46–0.57	0.000
Private rent	0.97	0.83–1.14	0.707	0.80	0.71–0.90	0.000
Constant	0.04	0.02–0.08	0.000	0.89	0.53–1.50	0.651
Pseudo R2	0.13					

Note: Reference groups are

1 Male

2 White

3 Married or in cohabitation

4 University degree

5 Managerial or professional

6 House owner.
